# Active learning through flipped classroom in mechanical engineering: improving students’ perception of learning and performance

**DOI:** 10.1186/s40594-021-00302-2

**Published:** 2021-07-22

**Authors:** Hyun Jin Cho, Kejie Zhao, Cho Rong Lee, Debra Runshe, Chuck Krousgrill

**Affiliations:** 1 Center for Instructional Excellence, Purdue University, West Lafayette, IN 47907 USA; 2 School of Mechanical Engineering, Purdue University, West Lafayette, IN 47907 USA

**Keywords:** Flipped classroom, Perceptions of learning, Academic performance, Mechanical engineering

## Abstract

**Background:**

To address some challenges that the large lecture-focused courses have faced in higher education, the flipped classroom model was implemented in mechanical engineering. The purpose of the study was to investigate mechanical engineering undergraduate students’ performance in the flipped classroom. A comprehensive analysis was conducted to investigate the pedagogical benefits of active learning in the flipped classroom from a self-determination theory perspective. To evaluate the effectiveness of the flipped classroom, students’ academic achievements in the flipped classroom were compared with the ones in the traditional lecture format. Moreover, to explore in-depth students’ learning experiences and their perceptions about the flipped classroom, students’ open-ended surveys were analyzed.

**Results:**

Results demonstrated that students in the flipped classroom performed better and favored the new model, feeling that flipped classroom was useful and helpful in preparing for the course. The qualitative findings showed that students felt that they benefited from the pre-week online lectures in the flipped classroom to prepare for the course.

**Conclusions:**

The current study shows that the flipped classroom model has the potential to create an autonomy-supportive learning environment and provide beneficial learning experiences. This study highlights the benefits of and future direction for implementing the flipped classroom in traditional mechanical engineering courses.

## Introduction

As undergraduate students’ enrollment in institutions has significantly increased across the USA, large classes that heavily relied on a lecture format have become more common throughout higher education (Cuseo, 2007; Toth & Montagna, 2002). However, the large lecture-focused courses have faced challenges, such as having higher withdrawal rates and impeding student engagement in course activities, which may be related to deficits in students’ academic achievement (Becker & Powers, 2001; Cuseo, 2007; Lax et al., 2017). To address these challenges, there has been an attempt to redesign or transform large-lecture courses to make a more active and student-centered environment (Hudson et al., 2014; Hudson et al., 2015). College instructors reorganize instructional design to improve students’ interaction, engagement, and academic achievement (Herrmann et al., 2003; Hudson et al., 2014). They incorporate effective pedagogical strategies, along with new  technologies, into their courses with large numbers of students to promote students’ learning experiences in traditional classroom settings (Boose, 2001; Graves & Twigg, 2006; Riffell & Sibley, 2004). These course redesign efforts provide a learning environment to improve student engagement and learning outcomes (Drab-Hudson et al., 2012; Fedesco & Cary,^ 2016^; Herrmann et al., 2003; Hudson et al., 2014; McLaughlin et al., 2014).

To respond to the recent paradigm shift from the traditional classroom to interactive course redesigned classroom, the flipped classroom model was implemented in the undergraduate curriculum in the school of mechanical engineering at a large Midwest university. The course was redesigned to improve students’ perceptions of learning and learning outcomes as part of the campus-wide course transformation program (Levesque-Bristol, Flierl, et al., 2019) that empirically examines whether the course design leads to desirable learning benefits and outcomes.

## Flipped classrooms in higher education

Engineering education should provide several essential pieces of training for students to develop problem-solving skills and substantial technical knowledge so that they can handle real-world issues in the workforce (Bishop & Verleger, 2013; Mason et al., 2013). However, faculty in engineering fields encounter several challenges in teaching a large-enrollment engineering class (Bongey et al., 2005; Herrmann et al., 2003). They might need to provide opportunities for students to actively engage with the material and to apply it to relevant fields, as well as to cover an extensive amount of teaching materials in class (Velegol et al., 2015). To address this challenge in a large-lecture classroom, a flipped classroom model has been applied in diverse engineering education fields (Davies et al., 2013; Dollar & Steif, 2009; Kaner & Fielder, 2005). According to the definition from several scholars, the flipped classroom is a pedagogical model where learning content is presented by individual online instructions prior to in-class meetings; and face-to-face, in-class time can be used for interactive group learning tasks and active learning (Albert & Beatty, 2014; Baker, 2000; Bishop & Verleger, 2013; Bland, 2006; Kerr, 2015; Strayer, 2012; Thai et al., 2017; Thorne, 2003). Because the individual online instruction is a supplementary session to support in-class interactive time, the flipped classroom model builds a mixture of online lectures and traditional face-to-face in-class time, which highlights learner-centered instruction (Gilboy et al., 2015; Thai et al., 2017).

The flipped classroom model has been widely applied in higher education, and the body of research regarding the flipped classroom is growing in various fields (Albert & Beatty, 2014; Bishop & Verleger, 2013; Kerr, 2015; Mason et al., 2013; O’Flaherty & Phillips, 2015; Simpson & Richards, 2015; Thai et al., 2017; Yough et al., 2019). The distinct characteristics of the flipped classroom to promote active learning were discussed in previous research on higher education (Fulton, 2012; Gilboy et al., 2015; Mason et al., 2013; Thai et al., 2017; Velegol et al., 2015). First, a flipped classroom adopts web-based and pre-recorded lectures to complement face-to-face time in the classroom-lecture setting (Thai et al., 2017). The pre-recorded lectures offer students more flexibility to learn new concepts or materials and to review fundamental concepts at their own pace and on their own time (Fulton, 2012; Johnston et al., 2013; McLaughlin et al., 2014). For this reason, students view the flipped classroom favorably (Fulton, 2012; Velegol et al., 2015). Also, once videos are recorded, these materials and classroom activities can be used in future classes (Mason et al., 2013). This type of online lecture is used as a complement to traditional face-to-face instruction that offers students the opportunity to review challenging concepts and catch up with missed classes (Johnston et al., 2013).

Moreover, because students learn fundamental concepts before attending classes, they are encouraged to engage in deeper learning through interactions during the in-class time (Gilboy et al., 2015; Kanelopoulos et al., 2017). In a traditional classroom where the course contents are delivered mainly by the instructor, students hardly interact with course materials or their peers during the class (Velegol et al., 2015). In the flipped classroom, however, because the course contents are delivered via online prior to the in-class time, students can have more face-to-face interactions with the instructor or peers and receive more individualized feedback during classroom activities (Zappe et al., 2009). Thus, students are more likely to actively engage in the course activities rather than passively listen to didactic lectures. Moreover, the flipped classroom allows instructors to apply various interactive teaching formats (Lage et al., 2000; Zappe et al., 2009). As a result, flipped classrooms offer a platform where students engage in active learning exercises in class such as problem-based learning and group work (Fulton, 2012; Mason et al., 2013; McLaughlin et al., 2014). Instructors in flipped classrooms can give immediate feedback to students while students actively work in learning activities requiring higher levels of Bloom’s cognitive taxonomy (Gilboy et al., 2015). Because students receive more individualized feedback while engaging in those activities in class, they can benefit from this interactive environment (Velegol et al., 2015). Previous research has shown that students in the flipped classroom were more likely to engage actively with the learning process and enjoyed interacting with faculty and peers during class time (Herried & Schiller, 2013; Velegol et al., 2015).

More importantly, the flipped classroom improves learning. The flipped classroom model has a positive effect on self-efficacy and intrinsic motivation (Thai et al., 2017). This format fosters students’ engagement and enables students to be prepared for higher-order academic activities such as problem-solving (Bishop & Verleger, 2013; Gilboy et al., 2015). Furthermore, this teaching approach has a positive impact on the accomplishment of student learning outcomes compared to a traditional classroom (Albert & Beatty, 2014; Roach, 2014). Some studies have shown that students in the flipped classroom demonstrate better academic performances than those in the traditional classroom (Lax et al., 2017; Mason et al., 2013). However, considering the mixed results regarding the effectiveness of the flipped classroom model (e.g., Yough et al., 2019), more research is needed on actual students performance to investigate the effectiveness of flipped classroom models (Kim et al., 2014).

Finally, the flipped classroom provides benefits to instructors, giving them a new role in the active classroom. Because students learn technical knowledge through online videos prior to the class, instructors can spend less time presenting technical content, allowing more class time dedicated to exercises and problem solving (Velegol et al., 2015). During in-class activities, the instructor acts as a facilitator or a guide while students complete the in-class interactive activities and provides additional assistance if students ask for help (Herried & Schiller, 2013; Velegol et al., 2015).

However, the existing literature reveals that it is limited in its discussion of methodological and theoretical perspectives on the flipped classroom (Karabulut-Ilgu et al., 2018). Previous literature has primarily focused on students’ satisfaction and perception of the class. More research is needed to assess students’ actual performance in the flipped classroom (Kim et al., 2014). Also, research needs to include more students’ voices and opinions to reflect their perceptions of the flipped classroom. Moreover, there is a need for instructors to implement the flipped approach in engineering courses. Research should aim to provide some specific guidelines to other instructors who are interested in implementing the flipped classroom approach. More importantly, the research on flipped classrooms needs a theoretical framework to explain why implementing the flipped approach works in large-lecture engineering courses. Our study bridges the gaps in current research.

## Self-determination theory

Self-determination theory (SDT) provides a theoretical framework to guide the course transformation in mechanical engineering courses (Deci & Ryan, 2002; Ryan & Deci, 2017). The course redesign was aimed to create a student-centered and autonomy-supportive learning environment where students take the initiative and actively engage in their own learning (Levesque-Bristol, Flierl, et al., 2019; Levesque-Bristol, Maybee, et al., 2019). SDT supports the idea of an autonomy-supportive learning environment where students’ basic needs are met and their autonomous motivation increases (Deci & Ryan, 2002; Ryan & Deci, 2017). According to SDT tenets, to empower individuals’ optimal psychological development, growth, and well-being, three different types of basic human psychological needs should be fulfilled: autonomy, competence, and relatedness (Deci & Ryan, 2002; Ryan & Deci, 2017). SDT (Ryan & Deci, 2017) suggests that when students are given choices or options in the learning process, they become autonomous (autonomy). Also, students can feel competent when they believe that they are able to deal with challenges in tasks and develop skills that they are expected to achieve (competence). Finally, when students get involved in caring relationships in the learning process, they feel connected to one another (relatedness). Students’ experiences in a learning environment that support students’ autonomy, competence, and relatedness are more likely to produce desirable learning outcomes such as intrinsic motivation, subjective well-being, higher achievements, and adaptive learning approaches (e.g., Levesque et al., 2004; Ryan & Deci, 2000, 2017).

Reeve (2006) introduced what an autonomy-supportive learning environment looks like in a classroom. In this type of learning environment, instructors promote students’ inner motivational aspects so that they are more likely to feel autonomous and competent in dealing with challenging tasks and connected with their peers and instructors, which, in turn, foster their autonomous motivation (Reeve & Jang, 2006; Ryan & Deci, 2000). Autonomy-supportive environments provide the instructional tasks around students’ inner motivational resources and informational feedback to achieve learning objectives (Reeve, 2006, 2009). More importantly, instructors with autonomy support attempt to structure a learning environment wherein students clearly understand what they are expected to do (Reeve, 2006). They have freedom in their choice in learning tasks and work in their own approach with self-pacing and receiving adequate assistance when they get stuck in the learning process (Reeve, 2006, 2009; Reeve & Jang, 2006). Numerous studies showed that the autonomy-supportive environments were significantly associated with students’ positive learning experiences such as autonomous motivation, greater involvement, more persistence, and learning outcomes (Haerens et al., 2015; Jang et al., 2010; Vansteenkiste et al., 2012).

In this study, the flipped classroom model is assumed to contribute to creating an autonomy-supportive learning environment. First, the flipped classroom allows students to learn with self-paced online videos so that they can work on their own (McLaughlin et al., 2014). As the online materials can be used at their pace and at their preferred time, the flipped classroom meets students’ autonomy and competence needs. Moreover, because the flipped classroom enables students to spend more interactive in-class time, it creates scaffolding where students can receive more personalized feedback during in-class tasks, nurturing students’ competence and relatedness needs (Yough et al., 2019). Besides, instructors can arrange the in-class interactive activities around students’ interests and preferences to support their autonomy (Reeve, 2009) as students learn the technical or fundamental knowledge before the in-class time. This in-class time enables students to actively engage in learning tasks and become active learners. As a result, it is expected that students’ academic achievement would improve in this autonomy-supportive and active learning classroom environment, as opposed to a passive one.

## Implementation of flipped classrooms in a mechanical engineering course

### Study context

The flipped classroom was piloted in a mechanical engineering class at a large Midwest  university. This course studies the statics of deformable materials upon applying external mechanical forces and/or thermal load. The key elements of the class include stress and strain in machine elements; mechanical properties of materials; extension, torsion, and bending of members; thermal stress; failure analysis; and design of a variety of structures.

### Pre-lecture video

In the pilot session lectured by the co-author Zhao, pre-lecture videos were recorded and edited in Camtasia and uploaded to Blackboard. Each video was 10 to 15 minutes long and covered the concepts of the topics that would be discussed in the coming week. The face-to-face instruction was taught on Mondays, Wednesdays, and Fridays, with each session lasting 50 minutes. Students were asked to watch the recorded videos before the Monday class each week. Watching the pre-lecture video does not automatically equate to students’ learning. For this reason, the instructor provided a pre-quiz prior to the in-person class so that students would watch the video and check their knowledge through the pre-quiz. In this way, students engaged with the online learning materials as a preview and gained points for their final grades, an incentive to those who complete watching the videos. Furthermore, the topic and fundamental concepts in the course could be consistent over the years, so students can make use of these previous videos solely focusing on learning core concepts. The strength of a flipped classroom is that once students review the contents and concepts, they can come to the classroom with questions about the content or struggles in understanding the content and can interact with peer groups and instructors during the face-to-face in-class time. This teaching approach provides students an opportunity for students to make sense of the material for themselves, which can lead to improvement in student learning. Focusing on students’ understanding, these interactions would affect students’ quality of learning. Also, if necessary, the instructors can freely edit or revise the pre-lectures or add more new materials.

### In-class activities

In the flipped, face-to-face classroom, the instructor recapped the concepts from the videos, focusing on examples and practicing problem-solving as individual students or as groups of two to three students. Students were encouraged to work together on the course materials. The instructor facilitated the discussions throughout the problem-solving process. Half of the Friday class was dedicated to a group quiz when students could work together on a problem, but each student submitted individual work. The difficulty level of the group quiz was comparable to homework and exam problems to bridge the gap between the in-class practice and assessment for students. The instructor facilitated interactions between peers to solve problems and provided help when students had questions solving the quiz problem. During in-class activities, students had more opportunities to interact with content materials and receive more timely feedback.

### Grading

Grading consisted of pre-week quizzes, in-class quizzes, homework, and three exams (exams 1, 2, and 3). To make the classroom an active learning environment, students were supposed to watch pre-recorded weekly videos about the theory and concepts of materials to be covered in the following week. After watching the videos, students were required to take a low-stakes pre-week quiz at any time before the class. They had 30 minutes to take the quiz in one sitting, and two attempts were allowed. The pre-week quiz was a conceptual quiz designed to evaluate how well students understood the concepts. In addition to the pre-week quiz, there was an in-class quiz every week based on materials covered that week. Typically, one homework set was due every week, except for weeks during which exams were given. Each exam had a maximum score of 100 that was worth 25% of the total course grade, while final grade values were between 0.0 and 4.0. Final grades included all three exam scores, homework, pre-week quizzes, and in-class quizzes.

### Current study

The current study examines whether the flipped classroom model is associated with students’ learning outcomes and how students perceive this new classroom model. The purpose of the study is to investigate the impact of using a flipped classroom approach in a mechanical engineering course, with the hypothesis being that the flipped classroom improves students’ learning. When testing these relationships between classroom formats and students’ learning outcomes, we considered students’ gender and ethnicity (i.e., international vs. domestic) that may affect students’ learning outcomes. The following research questions were investigated:

1. Do mechanical engineering students’ learning outcomes differ between students in flipped classes and students in traditional lecture classes?
2. Do students’ watching time for pre-week online lectures relate to students’ learning outcomes in the flipped classes?
3. How do students perceive the flipped classroom in the mechanical engineering course?

## Method

### Participants

Participants were recruited from students in a mechanical engineering course. There are different subsets of sampling for each analysis. First, for the comparison between the control group and intervention group, in total there were 99 students in the traditional classroom (Fall 2014, *n*=46; Spring 2015, *n*=53), while there were 313 students in the flipped classroom (Fall 2015, *n*=69; Spring 2016, *n*=39; Fall 2016, *n*=81; Spring 2017, *n*=50; Fall 2017, *n*=74) in total. These samples were used for hierarchical multiple regression analyses  to examine whether there was a significant difference in students’ learning outcomes when comparing the traditional classroom and the flipped classroom. The majority of students were white (51.5%), followed by international students (35.4%), Asian American (5.8%), Hispanic/Latino (2.4%), mixed-race (2.2%), unknown ethnicity (1.9%), and Black or African American (0.7%). For the international student population, the survey did not ask for specific ethnicity but only asked for an indication as to whether the student was domestic or international. The institution is a large research-intensive university where the largest number of international students major in science, technology, engineering, and mathematics (STEM) disciplines. According to the Office of International Students and Scholars (2015), Chinese students ranked first in total enrollment while Indian students ranked second. In addition, students from South Korea, Taiwan, Malaysia, Indonesia, Colombia, Iran, Bangladesh, Pakistan, and Turkey enrolled in this institution. Most students were male (86.7%), while 13.3% were female.

Secondly, the analytic data from Spring 2016 (*n* = 39) and Fall 2016 (*n* = 81) was used to examine the relationship between students’ online video viewing time and performance. Domestic students (white = 60%, Asian American=7.5, mixed-race = 4.2%, Hispanic/Latino = 2.5%, unknown ethnicity = 0.8%, and Black or African American = 0.8%) made up 75.8% of participants, and international students made up 24.2%. 

Finally, for the qualitative analysis, 78 students’ open-ended responses were used from Fall 2019 to explore students’ vivid voices and learning experiences in the flipped classroom.

### Data collection

To assess students’ academic performance after experiencing the flipped classroom, their three exams and final grades—calculated from pre-week and in-class quizzes, homework, and three exams—were used. In the qualitative phase, the open-ended survey was used to collect students’ perceptions of learning and instructional method concerning the pre-week online lectures in the flipped classroom in Fall 2019. The protocol was devised by the research team. Students were asked about their opinions regarding the format, aspects of the method that they thought were useful and effective, and the challenges they experienced. The current study was approved by the Institutional Review Board (IRB).

### Data analysis

A series of hierarchical multiple regression analyses were performed to test (1) whether there was a difference in students’ learning outcomes between the flipped classes and traditional lecture classes, and (2) whether students’ watching time for pre-week online lecture related to students’ learning outcomes in the flipped classes. We adopted a hierarchical multiple regression model where we added the demographic variables and class format at each step. We started by adding gender identification in Step 1, ethnicity in Step 2, and finally the course formats in Step 3. Gender was coded “0” for males and “1” for females. Ethnicity was coded as “0” for domestic students and “1” for international students. The class format was coded as “0” for the traditional lecture classes and “1” for the flipped classes. We added the gender and ethnicity variable to explore whether students’ gender or their nationality predicts learning outcomes. Finally, (3) to analyze students’ written comments, the qualitative content analysis was used to find common themes among the perceptions of the flipped approach (Vaismoradi et al., 2013).

## Results

### Learning outcomes between the flipped classes and traditional lecture classes

In total, there were 99 students in the traditional classroom in Fall 2014 and Spring 2015, while there were 313 students in the flipped classroom from Fall 2015 to Fall 2017. The majority of students were domestic students (64.6%), while 35.4% of students were international students.

To test whether there was a difference in students’ learning outcomes between the flipped classes and traditional lecture classes, a series of hierarchical multiple regression analyses were performed on exam 1, exam 2, exam 3, and the final grade. Table 1 shows the results of the analyses.

**Table 1:** Hierarchical regression models for learning outcomes (*N* = 412)

	B	SE B	β	*R*^2^	Δ*R*^2^
**Exam 1**					
Step 1				.001	.001
Gender	1.19	2.45	.02		
Step 2				.001	.001
Gender	1.12	2.45	.02		
Ethnicity	− 1.06	1.74	− .03		
Step 3				.224	.223
Gender	.37	2.16	.01		
Ethnicity	.69	1.55	.02		
Classroom format	18.73	1.73	.48**		
**Exam 2**					
Step 1				.005	.005
Gender	3.19	2.33	.07		
Step 2				.005	.000
Gender	3.20	2.34	.07		
Ethnicity	.14	1.66	.00		
Step 3				.153	.148
Gender	2.62	2.16	.06		
Ethnicity	1.50	1.55	.04		
Classroom format	14.59	1.73	.39**		
**Exam 3**					
Step 1				.000	.000
Gender	− 1.12	2.78	− .02		
Step 2				.001	.000
Gender	− 1.08	2.79	− .02		
Ethnicity	.50	1.99	.01		
Step 3				.001	.000
Gender	− 1.07	2.80	− .02		
Ethnicity	.47	2.00	.01		
Classroom format	− .36	2.24	− .01		
**Final course grade**					
Step 1				.001	.001
Gender	.12	.15	.04		
Step 2				.002	.001
Gender	.12	.15	.04		
Ethnicity	.06	.11	.03		
Step 3				.007	.005
Gender	.12	.15	.04		
Ethnicity	.08	.11	.03		
Classroom format	.17	.12	.07		

Two-tailed test of significance: **p < .001

 In Step 1, the demographic predictor variable *gender* was analyzed. The results of the analysis revealed a model not to be statistically significant (*p* > .05). In Step 2, the predictor variable *ethnicity* was added to the analysis. The results of the analysis revealed a model not to be statistically significant (*p* > .05). Finally, in Step 3, the predictor variable *classroom format* was added to the analysis. The results of the analysis revealed a model to be statistically significant for exam 1 (*p* < .001) and exam 2 (*p* < .001). Students in the flipped classroom were more likely to report higher scores on exam 1 (*M*_*trad*_ = 58.69 vs. *M*_*flipped*_ = 77.35, β = .48, *p* < .001) and on exam 2 (*M*_*trad*_ = 58.05 vs. *M*_*flipped*_ = 72.54, β = .39, *p* < .001) than those in traditional lecture classrooms.

For exam 1, adding *classroom format* to the regression model explained an additional 22.3% of the variation , and this change in *R*^2^ was significant: Δ*F* (1, 408) = 117.32, *p* < .001. For exam 2, adding *classroom format* to the regression model explained an additional 14.8% of the variation, and this change in *R*^2^ was significant: Δ*F* (1, 408) = 71.25, *p* < .001. The demographic variables *gender* and *ethnicity* were not significant predictors of any learning outcomes.

### Relationship between online pre-lecture videos and students’ academic performance

Online video viewing time refers to how many minutes students spent watching pre-lecture videos in total over the semester. To examine the relationship between total viewing time and students’ academic performance, data from 120 students in Spring 2016 and Fall 2016 was used.

First, the relationships between online lecture viewing time and each exam score were examined. Correlation analysis revealed that the online video viewing time before exam 1 and 2 was significantly correlated with exam scores. Exam 1 scores were correlated with online video viewing time before exam 1 (*r* = . 19, *p* < .05), exam 2 scores were correlated with online video viewing time before exam 2 (*r* = . 20, *p* < .05), and final grades were correlated with online video viewing time before exam 2 (*r* = . 22, *p* < .05) and exam 3 (*r* = . 24, *p* = .01). However, exam 3 scores were not correlated with online viewing time before exam 3 (*r* = .04, *p* = .68). One of the reasons could be that exams 1 and 2 have covered the contents of a certain number of pre-lecture videos before each exam. However, unlike exams 1 and 2, exam 3 has covered not only the contents after exam 2 but also all cumulative contents over the semester that students covered before exam 3.

To test whether students’ watching time for pre-week online lectures relates to their learning outcomes in the flipped classes, another hierarchical multiple regression analysis was conducted with samples from Spring 2016 and Fall 2017 (*n* = 120) that were available for this analysis (see Table 2). In Step 1, the predictor variable *gender* was analyzed, and the predictor variable *ethnicity* was added to the analysis in Step 2. The results revealed these two models not statistically significant (*p* > .05). The *R*^2^ value of .006 associated with the Step 1 model suggested that gender accounts for 0.6% of the variation in the final grade, while the addition of ethnicity accounts for 2.9% of the variation in the final grade. Finally, when the predictor variable *total viewing time* was added, the result of  hierarchical regression analysis revealed a model to be statistically significant. Adding viewing time to the regression model explained an additional 3.9% of the variation in the final grade, and this change in *R*^2^ was significant, Δ*F* (1, 114) = 4.75, *p* = .031. When all three independent variables were included in stage three of the regression model, neither gender nor ethnicity was significant predictors of the final grade. Together, the three independent variables accounted for approximately 7% of the variance in the final grade.

**Table 2:** Hierarchical regression models for final grades (*N* = 120)

	B	SE B	β	*R*^2^	Δ*R*^2^
Step 1				.006	.006
Gender	.25	.29	.08		
Step 2				.029	.023
Gender	.24	.28	.08		
Ethnicity	.37	.23	.15		
Step 3				.068	.039
Gender	.23	.28	.08		
Ethnicity	.19	.24	.08		
Viewing time	3.03E-5	1.39E-5	.21*		

Two-tailed test of significance: *p < .05

### Students’ qualitative responses

The open-ended survey was administered to explore students’ perceptions of learning and instructional method regarding the pre-week online lectures in the flipped classroom. The survey items asked students to write down their general learning experiences, benefits, challenges, and suggestions for the online lectures in flipped classroom format. To explore students’ learning experiences, a qualitative content analysis was employed to analyze students’ open-ended responses (*n* = 78) in Fall 2019. To identify common themes, the co-authors read the written comments a couple of times to become familiar with the open-ended answers and generated initial open codes based on the first 15 responses by utilizing an inductive approach. The researchers then included additional codes when new themes emerged by discussing and reaching an agreement on coding. Finally, they utilized two coder-generated initial codes and searched for general themes, eliminating and combining categories. After continued revision and refinement, the process yielded the most important themes captured from students’ responses. Based on this distilled information, the results are described as follows.

#### Learning experiences

Students shared their general learning experiences in the flipped classroom. For the first question about whether students favor continuing to have the lecture video, in total 73 students favored the flipped format while five students did not. Table 3 shows the summary of these themes. The following major themes emerged from students’ written comments to explain their preferences: Good introduction to the course (*n* = 40); helpful to prepare for the class (*n* = 25); helpful as a review and overview (*n* = 18); and provides active learning opportunities in class (*n* = 12). Students found that the flipped classroom provided a preview and overview, which could help them prepare for the in-class group activities in the upcoming classes. In addition, students highlighted the flexible aspect that flipped classrooms offered in both time and learning approach.

**Table 3:** Students’ learning experiences in the flipped classroom

Initial codes	Number of references	Main themes
- Helpful as a preview	40	Good introduction to the course
- Helpful to prepare for the exam - Helpful to prepare for the class	25	Helpful to prepare for the class
- Good to retain information - Good way to review - Overview - Helpful to go over the main concepts	18	Helpful as a review and overview
- To provide more time to solve problems	12	To provide active learning opportunities in class
- Working on their pace - Watching multiple times - Always available	3	Being flexible in time and ways

#### Usefulness

Students shared their thoughts about how and in what specific ways the flipped classroom format was useful (see Table 4). The main theme illustrated that students perceived the pre-week online lectures as useful in covering the theory and concepts before coming to classes. Covering the theory that might be new or challenging to students was the most significant benefit of the online pre-lecture for students (*n* = 56). Students also mentioned that the instructional method in the flipped classroom provided a preview of content before class (*n* = 22). Students commented that a quiz associated with the online pre-lecture video was helpful (*n* = 16). Miscellaneous opinions held that the pre-lecture video was always accessible online to rewatch (*n* = 9) and that it was an appropriate format for learning (*n* = 8) and good to review a concept (*n* = 8).

**Table 4:** Students’ perceptions on usefulness of pre-week online lectures

Initial codes	Number of references	Main themes
- Covering the theory - Derivation - Application example questions - Conceptual explanation and derivation: contents/step-by-step explanation - Formula list at the end of the video - Focus on the broad concept - Explanation of theory - Cover relevant material - Explanation of term for each section	56	Covering the theory
- Preview of content/content provided before class - Background of the topic - Topic introduction - Quick introduction of equation and concept	22	Introduction of the course
- Quiz helps understand materials better - Quiz associated with video - Length: (e.g., around 20 min)/good length - Example problem: number of questions	16	Helpful quizzes associated with pre-lecture videos
- Always accessible to lecture - Able to rewatch the lecture video if missed - Good pace to learn	9	Flexible ways of learning
- Format (one set of videos per week) - Going through each chapter separately - Visual aspect - Able to earn points - Frequency - The amount of material	8	Appropriate format (format-related)
- Good to review a concept - Overview - To force to look over notes	8	Good ways to review
- Allowing more class time - Prepare for exams	4	To prepare for the class

#### Challenges

The authors asked students what was challenging in the flipped classroom. The major themes emerging from students’ written comments can be seen in Table 5. Comments were related to time-related issues for flipped classroom pre-lecture videos in that the videos were too long/time-consuming (*n* = 27). Students also mentioned quiz-related issues (*n* = 22). For example, it was hard for them to remember the content for quizzes. When a quiz was not easily digestible, they could not solve the problem within two attempts. Moreover, they sometimes felt that the quiz was not relevant to the lecture video or confusing when it went in-depth.

**Table 5:** Students’ challenges

Initial codes	Number of references	Main themes
- Too long/time-consuming - Finding time to watch	27	The time-related issue in pre-week videos
- Short attempts in quiz - Hard to remember for the quiz: remembering to do a quiz on time - Not relevant to quiz: video and quiz does not match - Cannot solve the problem right away/not being able to ask questions until lecture	22	Quiz-related
- Hard to identify the topic: fixing the title - No title - Audio recording needs improving - Due date - Thorough layout of information	6	Structure-related
- Difficult to understand/confusing when it goes in depth, not easily digestible - Focused on too much information - Too much theory	4	Content-related

#### Suggestion

What students mentioned most frequently was the content-related issue (*n* = 31) in the pre-lecture video. They asked instructors to cut down on derivation. They stated that it would be great to have more example questions and examples connected to content, more real-life examples, more conceptual questions, shorter conceptual questions, more summaries, and simpler overviews. Also, other comments were related to the quiz length (*n*=27). Some even suggested eliminating the quiz. Miscellaneous comments were about the structure-related issues (*n*=6): the students suggested adding a closed caption, including more illustrations and page numbers, and separating the derivation and concepts in the pre-lecture videos.

## Discussion

The current study explores the flipped classroom setting in a mechanical engineering course. A flipped classroom was implemented to create an autonomy-supportive learning environment and increase active learning in mechanical engineering. Students were expected to watch the pre-lecture videos prior to class while being required to engage in interactive problem-solving or inquiry-based activities during face-to-face in-class instruction. A comprehensive analysis was conducted to investigate the pedagogical benefits of the flipped classroom. Our findings demonstrated that students in the flipped classroom performed better on exams. Moreover, the qualitative findings showed that students felt that they benefited from the pre-week online lectures in the flipped classroom to prepare for the course. The current study supports our hypothesis that the flipped model may have a significant association with students’ learning outcomes and that students may benefit from the learning environment created by the flipped classroom: to provide a preview to prepare for the in-class activities, accessibility, and flexibility for students to learn on their own, and more group activities opportunities. The learning environment where the flipped classroom is applied in an autonomy-supportive way seems to contribute to the satisfaction of students’ autonomy, competence, and relatedness needs; thus, these experiences may result in increased academic achievement.

As students mentioned in the open-ended responses, the pre-week online lectures seem to enhance students’ learning. Hierarchical multiple regression analysis results show that the more time students spent watching the online videos, the more likely they were to score higher. Also, there was a significant correlation between the viewing time of the pre-week online lectures and learning outcomes. This finding is aligned with the recent work that watching recorded lectures has a significant link to students’ academic achievement (Hoult et al., 2020; Jensen et al., 2018). More importantly, the pre-week online lectures would provide language and academic assistance to those students who need additional academic support and resources. It is possible that students who might struggle with course content and need language or academic assistance can benefit more (Jensen et al., 2018). Further investigation is needed regarding the potential of flipped classrooms to help underprepared students or international students achieve academic success.

Another way to explain students’ enhanced learning outcomes in the flipped classroom is that this new model provides an opportunity for students to become active learners. Because students learn the basic content knowledge before they come to class, there are more opportunities to work on problem sets during the class, and they are more likely to engage in classroom activities. Flipped classrooms may provide opportunities for students to have more interactions with the instructor and among peers during in-class activities (Herried & Schiller, 2013; Zappe et al., 2009). Therefore, while students actively engage in interactive in-class tasks during face-to-face instruction, they can build up higher-order cognitive skills and engage in meaningful learning (Bishop & Verleger, 2013; McLaughlin et al., 2014). For these reasons, the flipped approach is useful when teaching students in large enrollment introductory sections (McLaughlin et al., 2014).

In addition to becoming active learners in the flipped classroom, students are more likely to manage their time, learning strategies and approaches, and pace, which may be associated with self-regulation (Mason et al., 2013). Students gain basic knowledge and learn at their own pace. Moreover, the low-stakes formative assessments allow students to keep track of their learning process. These opportunities in the flipped classroom may encourage students to equip themselves with self-regulation skills that may be useful for their future careers (Kerr, 2015; Mason et al., 2013).

There are some considerations to take into account when implementing flipped classrooms in engineering courses. A thorough design is needed, considering the course objectives and characteristics of course content (Yough et al., 2019). Instructors need to consider how to present the pre-week online lectures and what type of in-class activities would best suit their instructional design and course objectives (Jensen et al., 2018: Twigg, 2009; Velegol et al., 2015). Because the flipped classroom is not the only factor that guarantees students’ improvement, the way in which it is implemented, along with how the class is constructed to improve engagement and interaction, should be considered first. For example, for the pre-week online lectures, it would be beneficial to have check-in quizzes or to have students accomplish a short concept test based on the lecture contents so that students may prepare for the theory and technical content on a regular basis before engaging in tasks that apply the concept (Kerr, 2015). Although some students may be resistant to taking quizzes, it would be vital to check their comprehension before they come to the class. More importantly, before instructors implement the new model in the class, it is essential to provide explanatory rationales of the flipped classroom model through sufficient and clear communication with students so that students can understand what they need to and what benefits they can expect (Gilboy et al., 2015; Reeve, 2009; Reeve & Jang, 2006). When instructors explain the rationale of the new model and suggest clear goals that students are expected to achieve, they can use this new teaching method in an autonomy-supportive way.

## Implications

There are several educational implications found in the current study. First, flipped classrooms can be a solution model to apply to large lectures (Hudson et al., 2014; McLaughlin et al., 2014; Patterson et al., 2010; Velegol et al., 2015). Implementing the flipped classroom requires a significant amount of time to prepare (Mason et al., 2013). However, this approach can provide active and interactive components in the traditional lecture environment to foster students’ engagement even in large enrollment classes. Thus, it can be applied to large-enrollment engineering introductory courses to create a student-centered and autonomy-supportive learning environment for undergraduate students.

More importantly, the flipped classroom can be a potential teaching and learning model during and after the COVID-19 pandemic (Talbert, 2021). During the pandemic, face-to-face instruction has significantly decreased due to diverse safety issues, but instructors still need to interact with students to make sure that they are learning in a timely manner. The flipped format can serve as an excellent solution to enhance students’ academic learning outcomes and decrease anxiety during the emergency remote learning environment (Smith, 2020). For example, in a virtual-only setting, the pre-recorded lecture format will remain the same, but the interactive activities among students and instructors can be done in a synchronous online session. Instructors can still give immediate feedback to students in synchronous online meetings or face-to-face class time. As per students’ comments from this study, the frequent interaction between students and instructors through a synchronous session is likely to contribute to an increase in the relatedness of students in a stressful environment.

Another important implication is that this study provides the theoretical framework to explain why the flipped classroom can benefit students’ learning, which is based on SDT. The theory explains that the learning environment that the flipped approach generates can enhance students’ basic psychological needs through interaction and engagement in class, leading to increased academic achievement (Yough et al., 2019). Using the SDT, this study provides a theoretical foundation to explain the educational benefits of the flipped classroom.

This study also employs a mixed method using qualitative data to capture students’ perspectives on the flipped classroom and to show the beneficial aspects of the flipped classroom from diverse perspectives. More importantly, this study contains students’ voices and opinions to reflect their perspectives and gives directions on how to improve the flipped classroom. Because many studies rely only on self-reporting surveys, the use of more robust research methods such as the mixed method and quasi-experimental designs is recommended (Velegol et al., 2015). Our study fills this research gap. Using a mixed method sheds light on more diverse aspects of the flipped classroom in engineering education.

Furthermore, the present study provides an overview of flipped classroom guidelines by addressing explicit design elements. Our study can act as a guide on how to implement the flipped classroom in a large introductory course. It specifically describes how to make the pre-week lecture videos and how to implement the flipped classroom in a large lecture course and what educational benefits to expect. Thus, our study provides guidelines for instructors who are interested in piloting the flipped classroom.

## Limitations and future directions

There are a couple of limitations to this study, some of which have already been suggested. First, there may be other factors affecting students’ learning achievement in the flipped classroom that would be addressed along with the variables tested in this study. In addition, although there was a significant difference in students’ learning outcomes observed, the authors were not able to assess the students’ learning strategies, as the previous literature pointed out (Thai et al., 2017). Future studies need to investigate whether the new format influences the various learning strategies that students apply to mechanical engineering. In particular, the way in which the flipped classroom promotes students’ self-regulated learning strategies should be studied to ascertain the relationship between the two with large quantitative data sets in the future. Finally, based on the findings from quantitative and qualitative analysis, including variables from qualitative feedback as predictors in the regression model may provide further insights into the future study.

## Conclusion

The findings of the study are promising and encouraging. The current study shows that the flipped classroom model has the potential to create an autonomy-supportive learning environment and provide beneficial learning experiences that may promote active learning, self-regulation, and engagement. This study highlights the benefits of and future direction for implementing the flipped classroom in traditional mechanical engineering courses.

## Data Availability

Data will not be shared in public due to the characteristics of the data.
